# Mature Teratoma of Posterior Mediastinum in a Newborn: A Case Report

**DOI:** 10.7759/cureus.82818

**Published:** 2025-04-23

**Authors:** Sokayna Safadi, Wiam Timsahi, Mehdi Saadoune, Fatima Ezzahra Khayi, Abdellatif Daoudi

**Affiliations:** 1 Pediatrics, Agadir University Hospital Center, Agadir, MAR; 2 Radiology, Sous Massa University Hospital, Agadir, MAR; 3 Anesthesiology and Critical Care, Sous Massa University Hospital, Agadir, MAR; 4 Neonatology, Agadir University Hospital Center, Agadir, MAR

**Keywords:** mature teratoma, newborn, paediatric thoracic surgery, posterior mediastinum mass, respiratory failure

## Abstract

Teratomas are germ cell tumors that can appear in different parts of the body, most often in the ovaries or testes. Treatment depends on whether the tumor is mature or immature: mature teratomas are usually removed surgically, while immature ones often require chemotherapy as well. Teratomas located in the mediastinum, particularly in the back part (posterior mediastinum), are rare. These tumors are typically discovered by chance during imaging exams, and only a few cases cause noticeable symptoms.

In this report, we describe a rare case involving a 9-day-old female newborn with no prior medical issues, who was brought to the pediatric emergency department with acute breathing difficulties. Her condition required admission to the neonatal intensive care unit due to respiratory failure. On examination, she had signs of respiratory distress, including a Silverman score of 6/10, fast breathing, and low oxygen levels. A chest X-ray revealed a mass in the left side of her chest, causing a shift in the mediastinum. A CT scan of the chest confirmed the presence of a large mass with both solid and cystic components in the posterior mediastinum, suggesting a mature teratoma. The baby underwent successful surgery to remove the tumor. Pathology confirmed it was a mature teratoma, showing a mass made up of different tissue types but no glial tissue. Her recovery went smoothly, with no complications during follow-up.

This case underscores how unusual it is for newborns to have symptomatic mature teratomas in the mediastinum. It also highlights the importance of considering this diagnosis in infants with breathing problems and shows that early surgical treatment can lead to a good outcome.

## Introduction

Teratomas, also known as germ cell tumors (GCTs), are neoplasms composed of different tissues from the three germinal layers: the endoderm, mesoderm, and ectoderm, and they are either mature (benign) or immature (malignant) [1]. They may be mature (well differentiated relative to the germ cell layer) or immature when incompletely or not differentiated, mimicking fetal or embryonic tissue. Teratomas are frequently located in the gonads; the mediastinum is the most common extragonadal location, and they are often anterior and mature [2], with nervous system tumors being the most frequent posterior mediastinal tumors [1].

Two theories on the formation of GCTs exist: the embryonic theory and the germ cell theory. The first theory suggests that totipotent cells, capable of generating various tissue types, escape body control and develop locally in the mediastinum without migrating into the scrotum; whereas the second proposes that each germinal tumor derives from an embryoma, a germinal cell [1].

Infantile and neonatal revelation of mediastinal teratomas is rare [3]. The diagnosis is oriented by clinical and paraclinical evaluation, and confirmation is based on histology of the removed specimen [2]. They may cause complications such as compression of thoracic organs, rupture, among others. To the best of our knowledge, we describe the first published case of a posterior mediastinal teratoma revealed in a newborn with respiratory failure.

## Case presentation

A nine-day-old female newborn was admitted to a neonatal intensive care unit (ICU) for acute respiratory failure. Her history showed a non-consanguineous marriage, gravida two, para two, and vaginal delivery at a gestational age of 37 weeks + 4 days. No abnormalities were observed on prenatal follow-up, and no neonatal asphyxia was noted (although the APGAR score was not specified).

At admission, her weight was 2,800 g (25th percentile), height was 48 cm (42nd percentile), and head circumference was 34 cm (76th percentile). She presented with dyspnea without cyanosis. Her Silverman score was 6/10, respiratory rate was 72 c/min, and peripheral oxygen saturation was 91% in room air. Her heart rate was 130 bpm, capillary refill time was <2 seconds, and she had warm extremities. There was no edema of the upper extremities or swelling of the face.

We observed abolished breath sounds in the middle and lower zones of the left chest wall. The right chest showed good air entry. S1 and S2 heart sounds were deviated to the right; no murmur or gallop was detected, and peripheral pulses were of full volume. Screening for choanal and esophageal atresia was negative. No external malformation was detectable on clinical examination.

We proceeded with airway clearance and oxygen therapy via nasal prong at 1-2 L/min, which achieved normal oxygen saturation.

**Figure 1:**
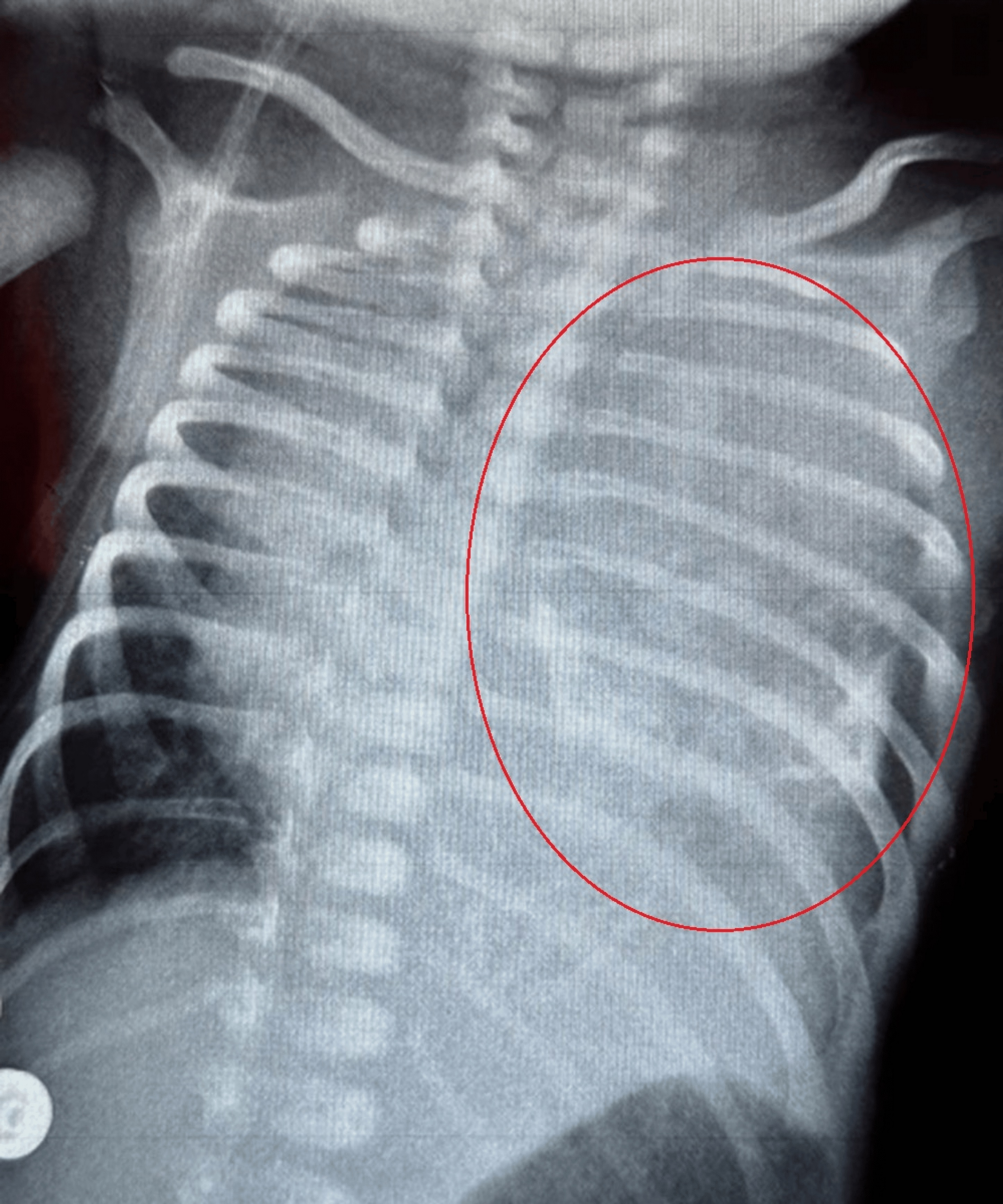
Chest X-ray showing a left-side intrathoracic mass The red circle shows the mass in the left hemithorax.

A chest radiograph showed a heterogeneous mass of the left hemithorax with mediastinal deviation to the right (Figure 1).

**Figure 2:**
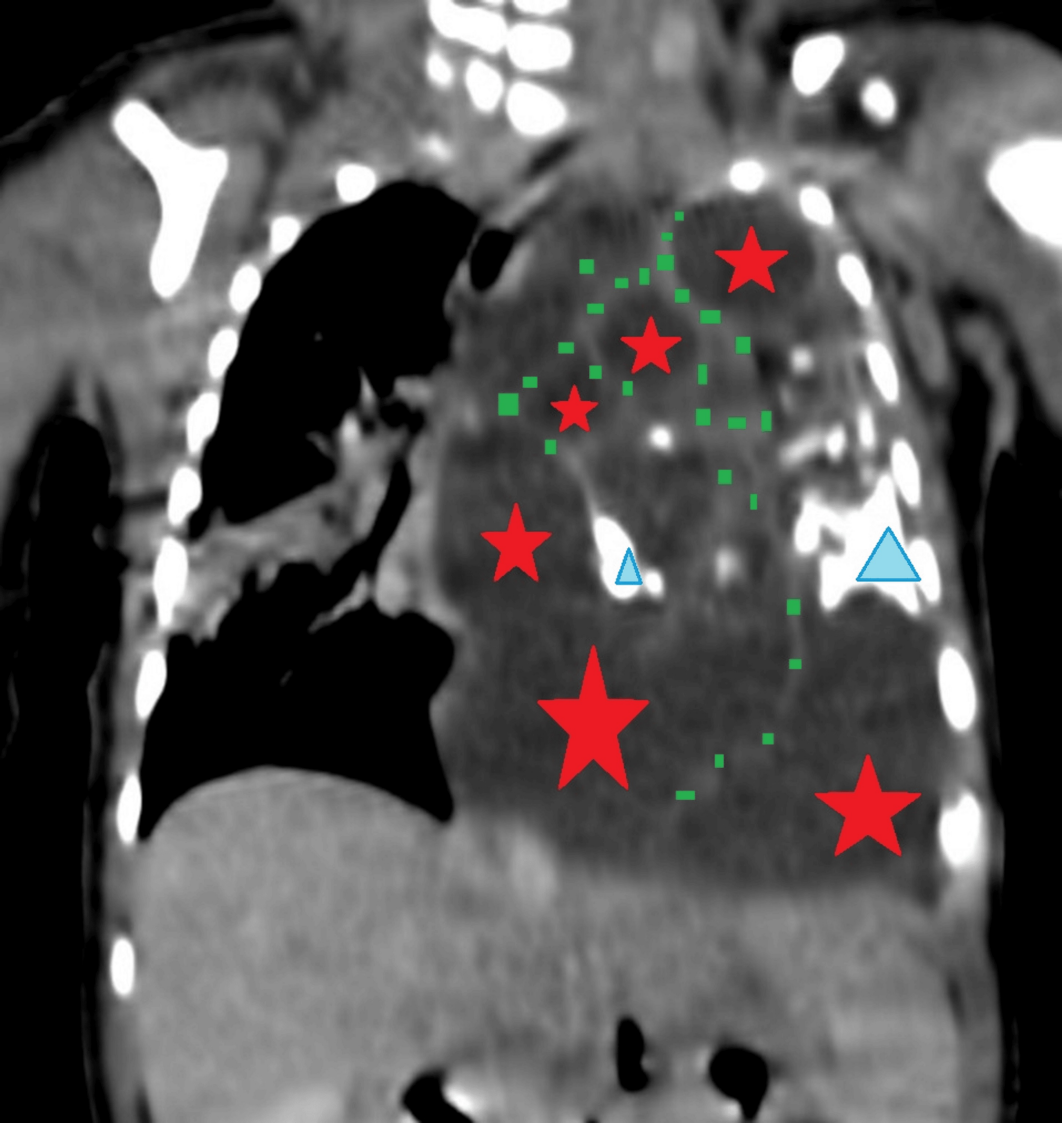
Injected thoracic CT scan objectifying the teratoma (coronal section) Injected thoracic CT scan at arterial phase in the mediastinal window demonstrating a voluminous mass with three components: cystic (red marks), tissue (green mark), and calcified (blue marks), associated with deviation of mediastinal structures toward the contralateral side.

**Figure 3:**
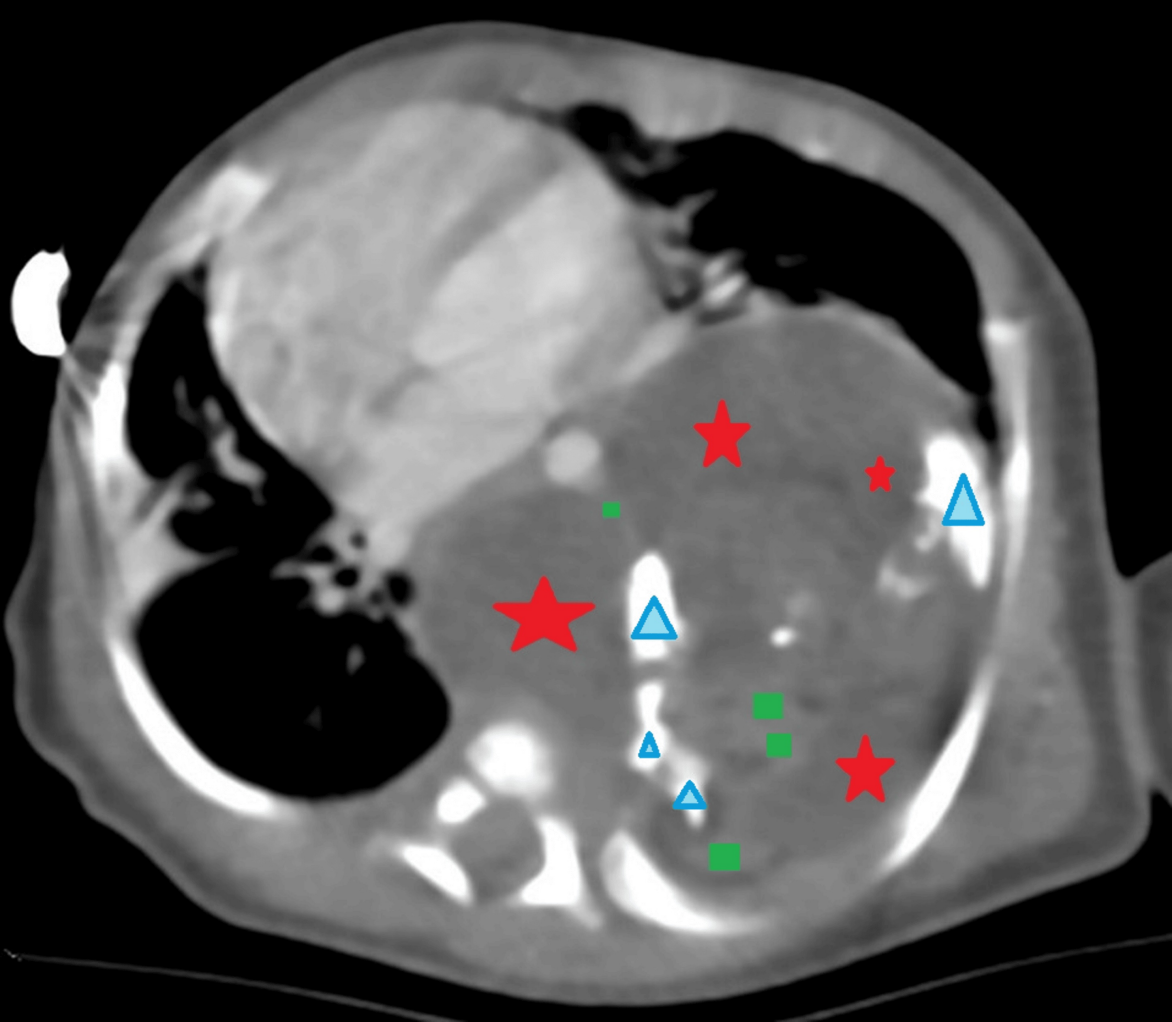
Injected thoracic CT scan objectifying the teratoma (axial section) Injected thoracic CT scan at arterial phase in the mediastinal window demonstrating a voluminous mass with three components: cystic (red marks), tissue (green mark), and calcified (blue marks), associated with deviation of mediastinal structures toward the contralateral side.

A thoracic computed tomography (CT) scan displayed a large solid-cystic mass measuring 58 × 54 × 71 mm in the posterior mediastinum, in favor of a mature teratoma, locally compressive on the left lung, with associated ventilation problems (Figures 2, 3).

**Figure 4:**
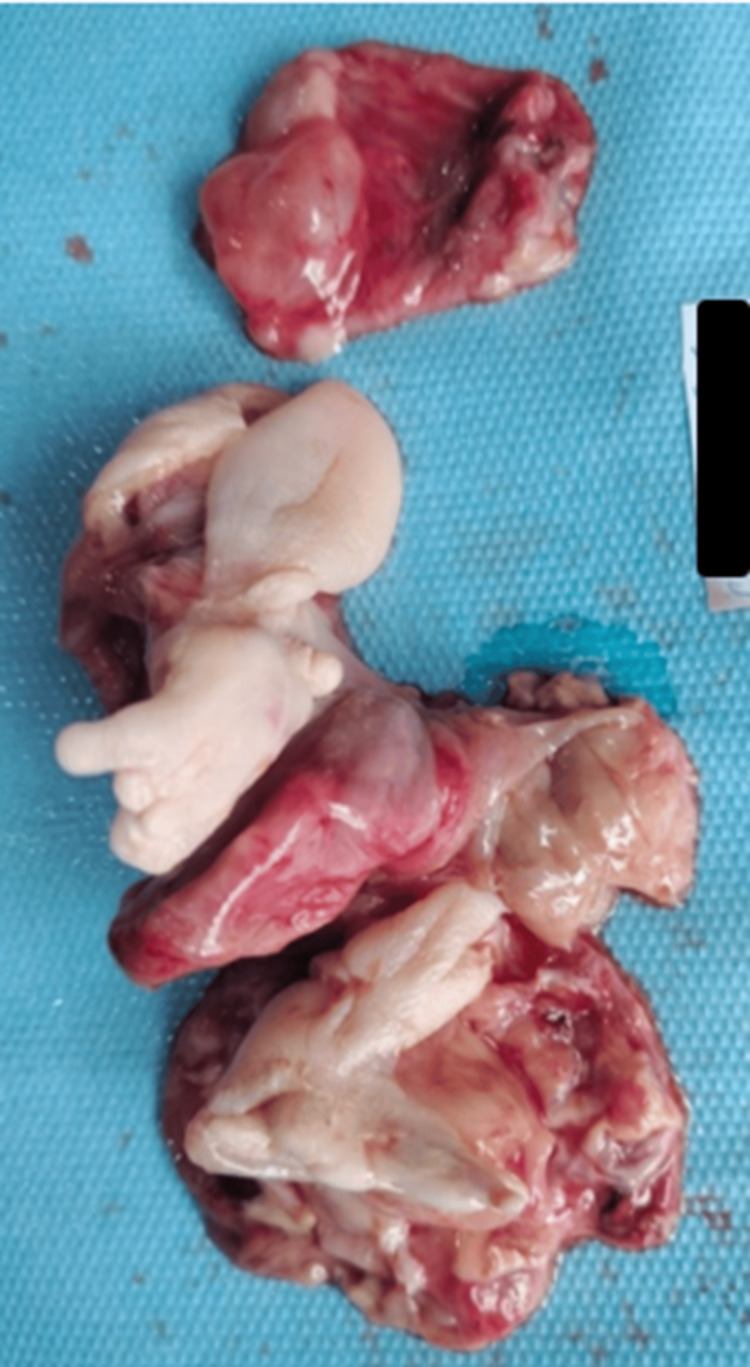
Macroscopic aspect of the removed piece Solid-cystic bilobed mass with teratomatous features, showing malformed membranes, bone, and a terminal segment of congestive appearance with cysts containing mucoid content.

**Figure 5:**
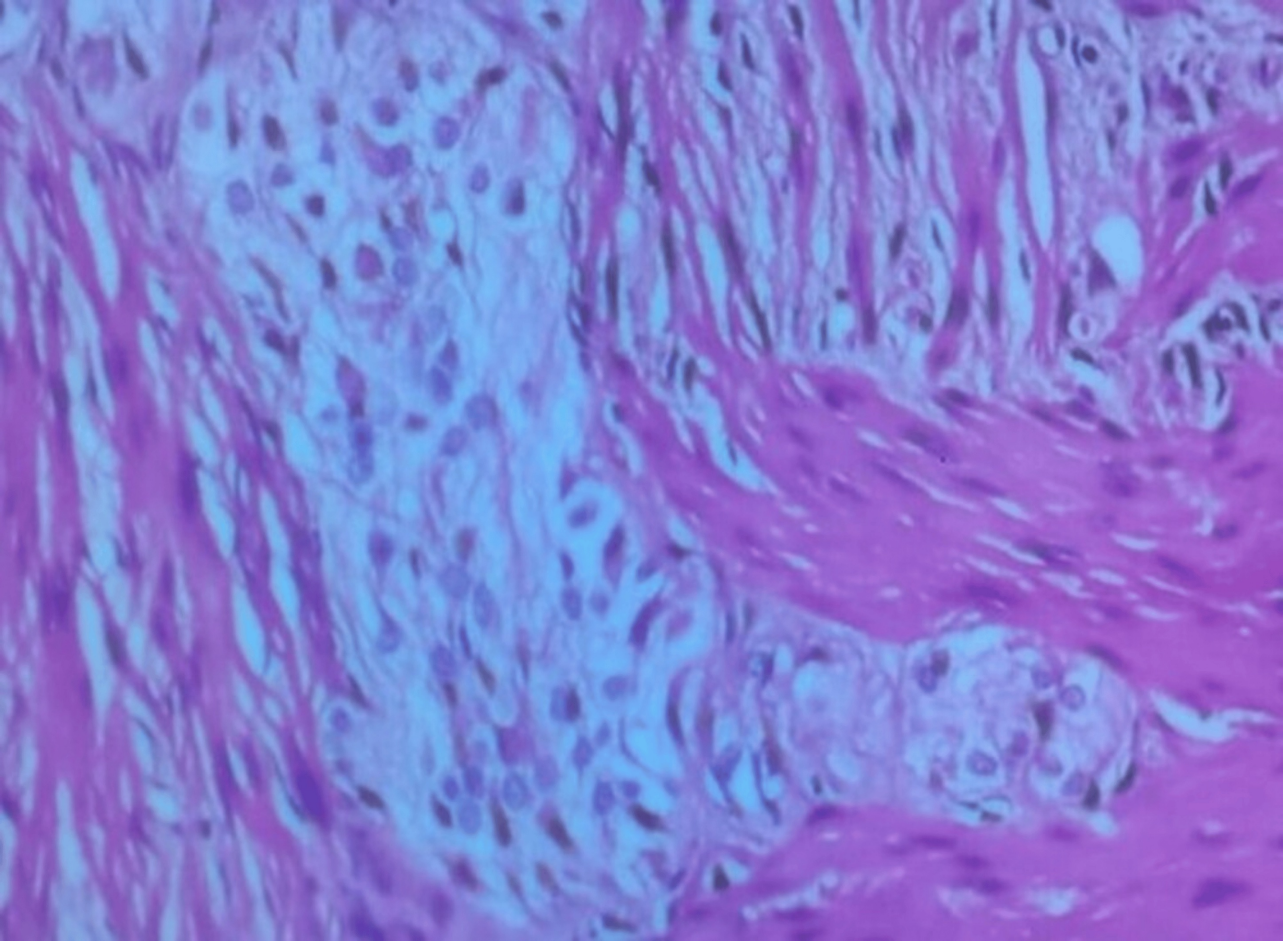
Histologic examination of the removed piece Pluritissular teratoma composed of various mature tissue types.

The patient was transferred to another center for surgery. She stayed in the ICU for 15 days due to superinfected bronchiolitis before undergoing successful surgery. The surgical procedure involved a thoracotomy with complete excision of the mass, which was closely adherent to most adjacent organs. Histopathological analysis of the tumor revealed a solid-cystic bilobed mass weighing 167 g and measuring 8 × 5 × 3 cm (Figure 4). It was identified as a mature pluritissular teratoma without glial substance. No malignant tissue was found (Figure 5).

**Figure 6:**
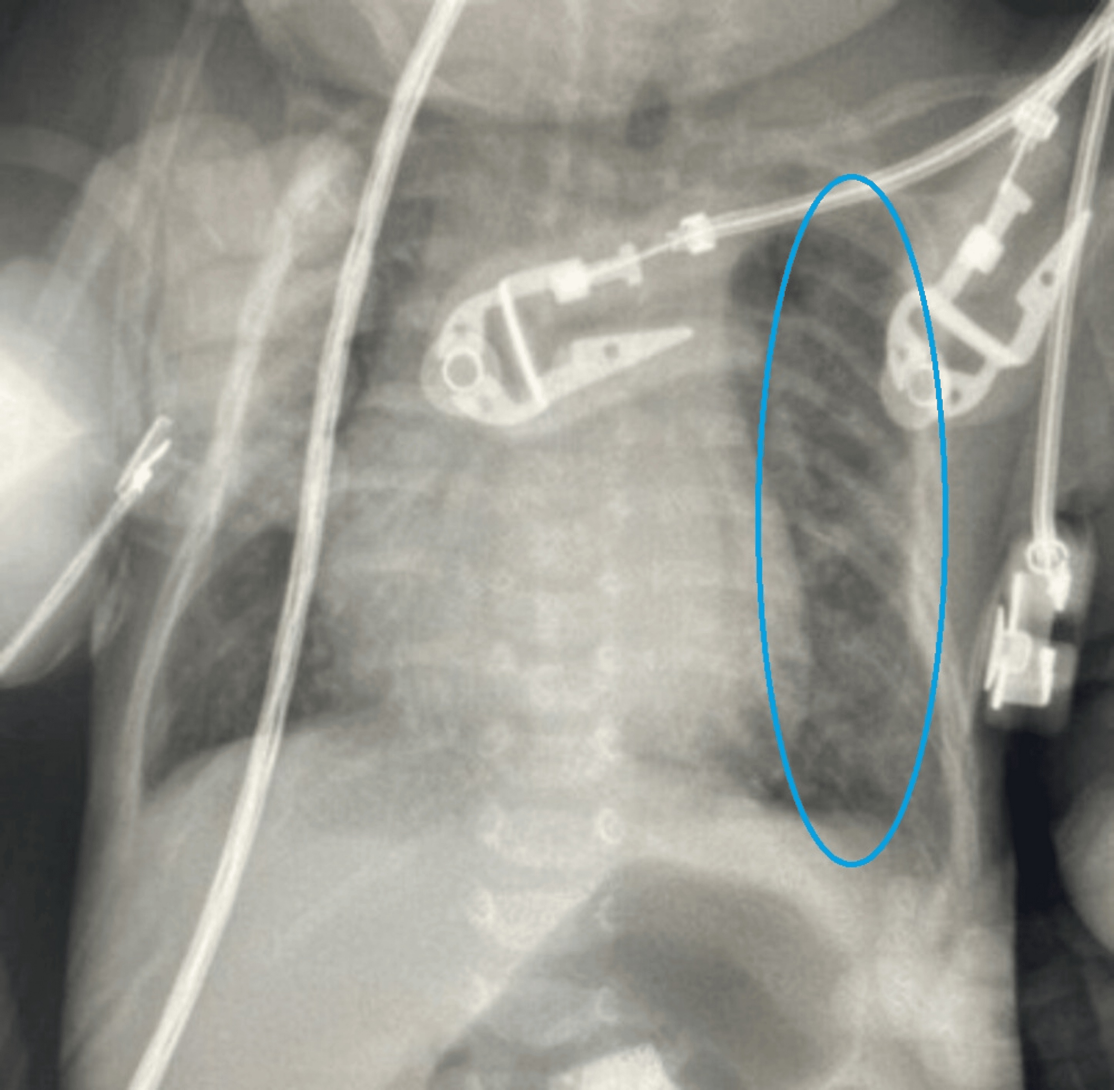
Postoperative radiograph The image shows absence of the mass (red circle) and return of the mediastinal structures to the midline position.

The postoperative recovery proceeded without significant events (postoperative radiologic control is shown in Figure 6). She was extubated three days postoperatively and discharged seven days later. She remained in good condition during the three-month follow-up and is scheduled to undergo follow-up evaluations every six months.

## Discussion

Germ cell tumors are congenital tumors arising from the germinal layers: endoderm, mesoderm, and ectoderm. They may be found in all organs, with a predilection for the reproductive systems and the sacrococcygeal region [3].

The mediastinal cavity is formed between the peritoneal, pericardial, and pleural cavities, emerging from the intraembryonic coelom. Mediastinal GCTs could arise from mediastinal totipotent cells that escape the physiological process and develop independently. These cells are capable of generating the different tissue types encountered in a GCT [1].

In the neonatal and infantile periods, the most commonly encountered teratomas are in the sacrococcygeal and presacral regions [4]. Gonadal teratomas are discovered at a later stage (testicular: under three years and peripubertal; ovarian: premenarchal) [5]. Mature mediastinal teratomas (MMTs) discovered early in childhood are rare, and even rarer in infancy or the neonatal stage [4].

MMTs often go unnoticed and are detected incidentally on chest X-rays [4]. A few case reports have described symptomatic MMTs discovered after a complication, mostly in adults [6-8]. Our patient presented at the age of nine days with acute neonatal dyspnea and desaturation. The symptoms worsened over time, indicating organ compression and requiring ventilator support.

A chest X-ray is the first paraclinical exam to investigate respiratory distress. Our patient’s radiograph showed a heterogeneous calcified mass in the left hemithorax, causing deviation of the mediastinum to the right. CT scans provide a better description of thoracic tumors and help guide diagnosis and therapy. Our patient’s thoracic CT scan displayed a large solid-cystic mass in the left posterior mediastinum, locally compressive, in favor of an MMT.

The standard treatment for an MMT is complete surgical removal. Surgery allows for definitive confirmation of diagnosis, corrects the symptoms, and prevents potential complications such as compression or rupture [4,7]. It is a major surgery, with possible complications such as phrenic nerve paralysis, recurrent laryngeal nerve paralysis, and pneumothorax [9].

Histological analysis establishes the final diagnosis of teratomas and determines whether they are mature or immature [2]. The composition of teratomas is generally heterogeneous, as they contain different materials such as fat, bone, hair, or other tissues. Our case confirmed a mature pluritissular teratoma without glial or immature tissue.

Unlike immature teratomas, mature teratomas are benign: complete removal allows full recovery without risk of relapse [10]. The postsurgical outcome in our patient was positive: the resection was complete, and she showed marked improvement in respiratory function. Because of her presurgical condition, recovery took longer, but she was asymptomatic at discharge. A follow-up at two months was unremarkable.

Our literature review found a few case reports on posterior MMTs in infants, but none in newborns. Mediastinal teratomas reported in infants were either anterior or immature. To the best of the authors’ knowledge, this is the first published case of a mature posterior mediastinal teratoma revealed during the neonatal period.

## Conclusions

A mature mediastinal teratoma represents a rare location for a germ cell tumor, with posterior placement being even more infrequent. Moreover, neonatal presentation of these tumors is exceptional. Imaging (X-ray and CT scans) guides the diagnosis, whereas a positive diagnosis and treatment rely on surgical removal. This article reports the rare case of a mature posterior mediastinal teratoma with neonatal presentation in a female patient, which necessitated surgical removal and showed excellent postsurgical recovery.
